# Business support and effects derived from COVID-19: implications on labor productivity

**DOI:** 10.47626/1679-4435-2022-719

**Published:** 2022-03-30

**Authors:** Erika Villavicencio-Ayub, Eliana Quiroz-González, Melissa García-Meraz, Erika Alejandra Santamaría-Plascencia

**Affiliations:** 1 Universidad Nacional Autónoma de México, Psicología, Ciudad de México, Ciudad de México, Mexico.; 2 Universidad Católica de Pereira, Psicología, Pereira, Risaralda, Colombia.; 3 Centro de Investigación, Dser Organizacional, Wollongong, New South West, Australia.

**Keywords:** coronavirus 2019-nCov infection, leadership, organizational productivity

## Abstract

**Introduction:**

The COVID-19 pandemic shows contagion and mortality rates that exceed the initial forecast and has caused a series of effects in different spheres of individuals, including the labor sphere.

**Objectives:**

To examine the relationship between business support and effects derived from COVID-19 in Mexican and Colombian workers and to identify the factors that can predict productivity.

**Methods:**

The sample included 482 people from both countries, 381 women (79%) and 101 men (21%). Two instruments were used: one scale to measure business support and the other to measure COVID-19 effects. A multivariate design was used to understand the factorial structure of the instruments through confirmatory factor analysis, and a predictive design was used based on structural equation modeling.

**Results:**

Statistical analysis was conducted through the confirmatory factor analysis and found a factorial solution that fitted the theoretical approach to the data in both instruments, and the structural equation model showed an adequate goodness-of-fit (*X*^2^ = 320.110, p = 0.000; comparative fit index = 0.90; root mean square error of approximation = 0.07).

**Conclusions:**

According to the results, it was observed that both instruments presented an adequate goodness-of-fit to the data. The structural equation showed that leadership (0,420) and daily disturbance (-0.558) are predictors of labor productivity. Specifically, a positive leadership style facilitated group performance and therefore the achievement of results. Daily disturbance, such as effects derived from COVID-19, negatively affects productivity; therefore, all actions taken by organizations to provide support will promote mental health and will thereby help to achieve the expected productivity.

## Introduction

The pandemic has affected not only the economic and social spheres but also mental health,^1-3^ leading to job losses^4^ and bringing important consequences both for organizations and for workers.^5^ To face this crisis scenario, organizations seek to find alternatives to survive in the immediate term and to implement strategic changes that allow for them to make long-term projections.^6^

Although this pandemic is global, some countries experienced greater effects, both due to the number of cases and deaths and to the impact derived from a disadvantageous social system, among other factors. According to recent data, countries such as Mexico and Colombia are among the ten countries with the highest number of COVID-19 infections in the world.^7^

The pandemic has left several lessons to be learnt, such as understanding that biological factors are essential to contain the virus, but psychological and social factors play a transcendental role and are those that turned the pandemic into a global disaster.^8^ It is thus crucial to understand psychological and social aspects; hence, work, labor relationships, and occupational health are the focus of relevant studies.

In this sense, the labor context has experienced major negative consequences derived from the pandemic, which has put the sustainability of millions of organizations to the test. Several sources of employment have been closed down, some others have migrated to remote work, essential services have remained in the frontline, in a constant effort that brought repercussions to workers either due to long working hours, blunders resulting from the lack of guidelines and policies, negative leadership styles, among other factors. Conversely, some work cultures facilitate work continuity with lower occupational burnout, thanks to their efforts in implementing strategies aimed at workers’ well-being and mental health care.

### Business support to work during COVID-19

Organizational culture and the production sphere pose multiple challenges both to entrepreneurs and workers. One of these challenges is that organizations make their processes more efficient and implement strategic actions to respond to the crisis, which involves, among other essential elements, identifying the leadership styles required in these times and that respond to the needs for efficiency and achievement of goals. Times of crisis highlight the importance of appropriate management of working teams, with leaders who provide clear directions, make optimal attribution of responsibilities, and make proper use of management skills, such as assertive communication, resilience, and innovation.^9,10^

Conversely, it was the first time that organizations implemented remote work to adapt to the unexpected confinement measures imposed to reduce virus spread. In this sense, millions of workers had to adopt this modality to continue to work.^11^

Remote work may become highly satisfactory when considering the time saved by not commuting and that could be spent in activities that were possibly neglected, including the opportunity of greater family interaction, achieving a better work-life balance. Workers who adapt to this modality with the monitoring of a leader and established policies, increase their work engagement, sense of belonging, quality of work, and other factors that affect business productivity.^12^

In turn, business support obtained in this management will make it easier to adopt remote work by establishing an appropriate relationship between the manager and employees, as well as constant and effective feedback, timely identification of emotional effects. Contact should be maintained regardless of the distance, making workers feel as a crucial element in the team.^11^

Productivity and work satisfaction are related to several aspects, and workers decide to remain in jobs where they have the opportunity of using their skills and have varied assignments, constant feedback, fair wages, among others. The positive relationship between both variables contributes for satisfied workers to become productive workers.^13^

Even though constant efforts have been made to establish occupational guidelines in several workplaces, the organizations that have the human element as the center that guides business sustainability.

### Effects derived from COVID-19

Effects caused by COVID-19 are multiple and involve daily life disturbances, concerns, and fears, among others. The Organization for Economic Co-operation and Development estimates a 2.4% decrease in global economic growth for 2020, because supply chains, raw material, loss of business confidence, and crisis in several sectors will be present; this decrease could lead to economic recession in some countries.^14^ This implies instability in global economy, with different repercussions for each country, since economic and political developments are heterogeneous, which interferes with national economies.^15^

Thereby, disturbances in daily life during the pandemic may cause important mental health sequelae that are partially associated with labor problems and job loss.^16^ Job loss has not only economic repercussions; it also affects the sense of life and well-being,^17^ because the space where work in carried out is not only a scenario of positive interchange and being understood but also a space of life^18^ where people are able to develop their skills and experience satisfaction for achieving their goals.

Therefore, COVID-19 has changed everyday life,^19^ causing fear in some people, related, for example, with the probability of contagion, disease, and death.^20^ Furthermore, difficulties that may be experienced due to health emergency, because different activities were moved to within households; which results in a longer stay in this place, with an opportunity to strengthen affective bonds or to increase conflicts between family member,^21^ the latter representing a risk for intra-familial violence.^22^ Additionally, those who remain at home during their working hours may have an additional risk factor derived from the expansion of their roles, which is translated into increased working hours,^23^ disrupting work-family conciliation and life balance.

In addition to the above, many stressors associated with this health emergency have led to an increase in suicide risk^24^ and in the consumption of opioids,^25^ of alcohol and tabaco,^26^ and of audiovisuals media,^27^ as well as to worsening of psychiatric disorders.^28^

In view of this need, the present study aimed to examine the relationship between business support and COVID-19 effects on Mexican and Colombian workers, as well as to identify the factors that can predict productivity. Precisely, this study seeks to provide evidence to support the decision-making of business directors addressing phenomena such as impacts on workers and business support and thus contribute to the improvement of occupational health and business sustainability.

## Methods

### Design

A correlational design was used to understand the factor structure of the instruments through confirmatory factor analysis (CFA). In the second stage, a predictive design was employed through structural equation modeling, in order to identify the relationship of leadership, business support, daily disturbance and predicted productivity.

### Sample

Total sample included 482 people, 381 women (79%) and 101 men (21%). Half of the sample came from Mexico, and half from Colombia. The sample was obtained through contact with working people by social networks and email. They were invited to participate and informed about the objective of the study (Table 1).

**Table 1:** Sociodemographic data of the sample

Variables	Mean (SD) Mexico	Mean (SD) Colombia
Sex		
Women	189 (78.4%)	192 (79.7%)
Men	52 (21.6%)	49 (20.3%)
Age		
18 to 23 years	23 (9.5%)	278 (11.2%)
24 to 40 years	153 (63.5%)	160 (66.4%)
41 to 55 years	58 (24.1%)	49 (20.3%)
56 years or older	7 (2.9%)	5 (2.1%)
No. of children		
None	132 (54.8%)	155 (64.3%)
1 to 2 children	96 (39.8%)	76 (31.5%)
3 to 4 children	11 (4.6%)	9 (3.7%)
5 children or more	2 (0.8%)	1 (0.4%)
Marital status		
Single	112 (46.5%)	135 (56%)
Married	41 (17.0%)	47 (19.5%)
Domestic partnership	78 (32.4%)	40 (16.6%)
Divorced	8 (3.3%)	16 (6.6%)
Widowed	2 (0.8%)	2 (0.8%)
Schooling		
Elementary school	7 (2.9%)	13 (5.4%)
Technical career	17 (7.1%)	12 (5%)
Undergraduate degree	116 (48.1%)	97 (40.2%)
Graduate degree	101 (41.9%)	119 (49.4%)
Type of house		
Own	153 (63.5%)	101 (41.9%)
Rented	49 (20.3%)	106 (44%)
Shared	39 (16.2%)	34 (14.1%)
Type of company		
Public	67 (27.8%)	64 (26.6 %)
Private	174 (72.2%)	152 (63.1%)
Did not answer	-	25 (10.4%)

### Instrument

Two scales validated for the concerned population were used.^29^ The first scale assesses business support to work during COVID-19 and consists of 16 questions in a 6-point Likert scale ranging from “Always” to “Never.” The scale is composed of three factors and one indicator: business leadership (34% of explained variance, alpha of 0.860), organizational support (11.64% of explained variance, alpha of 0.710), productivity (9.14% of explained variance, alpha of 0.710), and remote work (7.35% of explained variance, alpha of 0.492). The instrument explains 62.14% of the variance, with an overall reliability of 0.842.

The second scale measures effects derived from COVID-19 and is composed of 17 questions in a 6-point Likert scale ranging from “Never” to “Always.” The validation of this instrument was reported by exploratory factor analysis. The five factors included in the scale are: disturbance of daily life (31.69% of explained variance, alpha of 0.705), economic effects (11.43% of explained variance, alpha of 0.811), concern (7.81% of explained variance, alpha of 0.752), fear (6.24% of explained variance, alpha of 0.690), and occupational effects (5.92% of explained variance, alpha of 0.783). Overall, the instrument explains 61.3% of variance, with a reliability of 0.867.

### Statistical analysis

In order to understand the structure of the instruments, a CFA was conducted for each dimension; the analysis was conducted for categorical data, due to non-normal data distribution. Subsequently, structural equation modeling was employed to identify the variable that best predicts productivity in remote work. Statistical analyses were conducted using SPSS software, version 22, and R software 3.6.1.

### Ethical considerations

This investigation is in compliance with the Declaration of Helsinki standards. Informed consent was obtained. Furthermore, this study was approved by the research ethics committee of Universidad Católica de Pereira, as stated in protocol 092020.

## Results

### Confirmatory factor analysis

#### Business support to work during COVID-19

**Figure 1:**
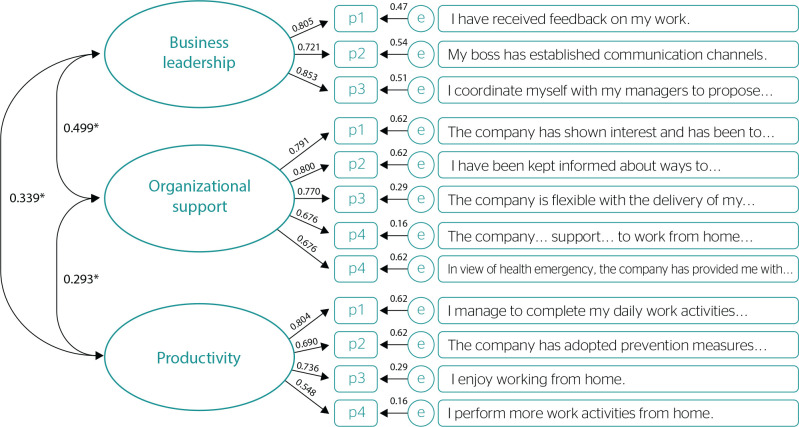
Business support for work during COVID-19. * p < 0,001.

Through CFA, a factor solution was found to fit the theoretical proposal to the data. The main indicators showed *X*^2^=3850.90, p = 0.00; comparative fit index (CFI) = 0.97; root mean square error of approximation (RMSEA) = 0.06, 95% confidence interval (95%CI) [0.49-0.75] (Figure 1).

#### Effects derived from COVID-19

**Figure 2:**
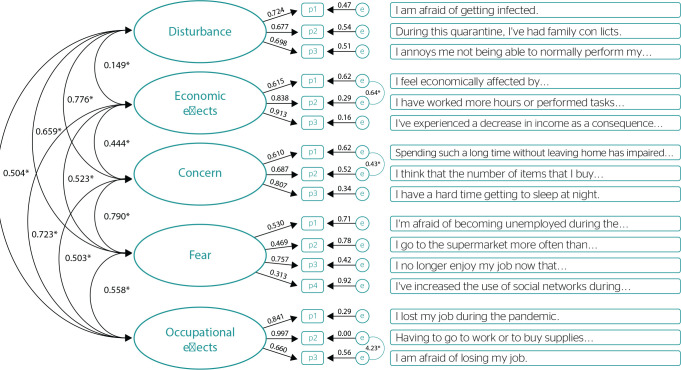
Confirmatory factor analysis of the COVID-19 effects scale. * p < 0,001.

Through CFA, a factor solution was found to fit the theoretical proposal to the data. The main indicators showed *X*^2^=331.51, p = 0.00; CFI = 0.97; RMSEA = 0.07, 95%CI [0.62-0.79] (Figure 2).

### Structural equation model

**Figure 3:**
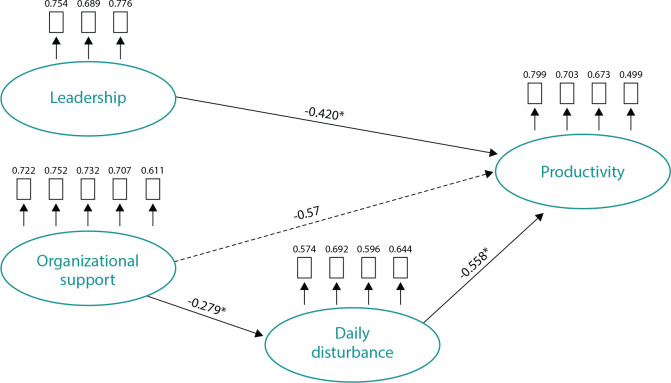
Structural equation model for COVID-19 and productivity. * p < 0,001.

Productivity is predicted by leadership, but not by organizational support. Interestingly, productivity is negatively predicted by Daily disturbance; however, Organizational support negatively predicts Daily disturbance. Thus, organizational support may contribute to prevent daily disturbance and improve productivity. The model showed appropriate goodness-of-fit (*X*^2^=320.110, p = 0.000; CFI = 0.90; RMSEA = 0.07) (Figure 3).

## Discussion

According to study results, it was possible to observe that the two instruments showed appropriate goodness-of-fit to data. The first instrument, related to Business support, is composed of dimensions concerning Leadership, i.e., the perception that the leader establishes a communication, plans strategies, and organizes employees’ assignments. However, this instrument also includes a question concerning on leaders and coworkers who communicate with employees, even outside usual working hours, which affects life balance and prolongs the time during which workers are dealing with work issues.

The second dimension of COVID-19 effects encompasses the daily life disturbances resulting from confinement. It is important to mention that this dimension encompasses a series of situations such as family problems, work conflicts, fear of contamination, and annoyance for not being able to perform everyday activities outside home. This dimension is particularly important in the overall configuration of the instrument.

Structural equation showed relevant data: productivity is predicted by leadership (0.420) and by daily disturbance (-0.558), but not by organizational support (-0.057). Interestingly, companies that provide support in terms of flexibility and interest do not have a direct impact on labor productivity, understood as the achievement of work goals with work satisfaction. However, company support has an impact on daily disturbance (-0.279). This result clearly indicates that actions of company support could have an indirect impact on workers’ productivity by mitigating disturbance, fear, and family conflicts, so that to experience greater satisfaction with performing their tasks from home.

One way to reduce this disturbance could be associated with building positive social relationships, because it contributes to personal and professional development and favors coping with adverse situations; in this regard, companies could design and implement healthy organizational practices,^5^ as a mechanism to promote workers’ mental health.

It is necessary to accept that the pandemic has caused a global change^29^ and that, consequently, companies, their policies, guidelines, and especially the role of human aspects in work organizations should be transformed. Therefore, in crisis times, there is an urgent need to search for strategies to promote workers’ quality of life and organization resilience, so that to also strengthen business structures that allow for greater competitiveness.^5^

This health emergency should be faced with disciplinary and professional resources;^22,30^ therefore, the results of this investigation are expected to provide support for organizational decision makers to review leadership styles and the support that they provide, as an institution, to their working teams. This should be considered as a practice of social responsibility and of human sense and as a strategic action, given its implications for productivity.

There are many challenges to be included in the work agenda, such as discovering new perspectives, theories and methodologies that explain the processes of crisis but, above all, allow for broadening knowledge on the ways to cope with adversity using personal, organizational, and social resources.

## Conclusions

Phenomena such as the pandemic provide the opportunity de measure, assess, and propose constructs in organizational systems that improve labor productivity without neglecting employees’ quality of life. Both variables are key for organizations to remain in this competitive market even in times of crisis. In this sense, the present investigation provides reliable measuring tools for the study population and opens the possibility for additional studies to obtain evidence on the psychosocial characteristics of both countries.

In this context, and according to the findings of the present investigation, business support, and thus the dimension associated with leadership, and COVID-19 effects, especially daily life disturbance, predict labor productivity among Mexican and Colombian workers. Therefore, it is essential to strengthen the inside of companies and the actions aimed at a fostering business support, leadership, and workers’ health care in their daily activities, because these factors have an impact on workers’ productivity.

One of the limitations of this study is related to its sample size. We recommend that the measuring instruments used herein, as well as other forms of measurement and assessment, continue to be applied to provide a deeper understanding of the consequences of social distancing, working from home, and business policies. Furthermore, it is necessary to conduct additional studies that make it possible to differentiate business support or policy and workers’ well-being. Future investigations will be able to deepen the issue of what working from home means, respecting working hours and the right to disconnection from work.
